# Comparison of Illness Concepts and Coping Strategies among Cancer Patients of Turkish and German Origin

**DOI:** 10.3390/ijerph17155580

**Published:** 2020-08-02

**Authors:** Katja Thein, Yesim Erim, Eva Morawa

**Affiliations:** Department of Psychosomatic Medicine and Psychotherapy, University Hospital of Erlangen, Friedrich-Alexander University Erlangen-Nürnberg (FAU), 91054 Erlangen, Germany; katjathein@yahoo.de (K.T.); yesim.erim@uk-erlangen.de (Y.E.)

**Keywords:** illness concepts, illness beliefs, coping strategies, cancer, Turkish, migrants

## Abstract

Background: The aim of this study was to compare illness concepts and coping strategies among native German cancer patients and those with a Turkish migration background. Methods: Guideline-based, semi-structured interviews were conducted with 11 German (♂: 8, ♀: 3) and 11 Turkish (♂: 2, ♀: 9) cancer patients. The transcripts were evaluated using a qualitative content analysis in accordance with Mayring. Results: We identified eight categories of illness concepts: stressful life events, environmental influences, the will of God, medical factors, fate, trauma, health behaviour, and psychological causes. German patients frequently attributed their illness to environmental influences, persistent stress, or medical factors, whereas Turkish patients blamed persistent stress, the will of God, or trauma. The last two categories are not found among German patients. We classified the coping strategies into 11 main categories: social support, activity, patient competence, fighting spirit/positive thinking, use of health services/alternative healing methods, lifestyle, emotional coping, cognitive coping, religious coping, spiritual coping, and culture-specific methods for patients of Turkish origin. For German patients, activities as well as social support played primary roles in coping. Turkish patients also often used social support. However, in contrast to the German patients, they are less active and use much more religious coping and culture-specific means. In addition, negative emotions occur more often when processing the illness than in the German patients. Conclusion: Common illness representations and coping strategies could be found for Turkish and German patients, but also specific ones for the respective group. It is particularly noticeable that German patients attach more importance to medical factors and try more actively to cope with the illness. For Turkish patients, cultural and religious factors play an important role, which should also be considered in treatment.

## 1. Introduction

Cancer is the second most frequent cause of death in Germany [1], and the annual incidence is about 492,000 [2]. Men are most often affected by lung cancer, followed by colon and prostate cancer. For women, breast cancer is first, lung cancer second and colon cancer third among the entities [3]. When confronted with the diagnosis of cancer, many patients may question the meaning of their lives and feel that they no longer have any control over their lives. The diagnosis frightens patients and relatives and is even the most feared diagnosis in Turkey [4,5]. Patients try to find explanations, and this leads to so-called “subjective concepts of illness” often emerging. These can be described as the sum of all opinions, interpretations, explanations, and predictions concerning disorders of a person’s state of health [6]. These concepts have a complex structure [7] and serve to form the way a person handles an illness. In this context, the affected person develops individual coping strategies to maintain a certain quality of life for as long as possible. “Coping” is defined as cognitive and behavioural efforts of a person used to deal with stressors and stressful situations [8].

In the context of an illness, cultural influences should always be taken into account. This has been postulated for mental illnesses in many cases [9], but cancer is also influenced by cultural factors [10]. Bermejo et al. examined Spanish and German lay theories on the development of myocardial infarction and cancer, whereby internal attributions dominated in German patients, while Spanish patients tended to hold onto fatalistic belief systems [7]. Franz et al. draw a comparison of the concepts of mental illness between Turkish and German patients, with German patients primarily developing internal concepts of illness and becoming actively involved in the illness process. Turkish patients, on the other hand, tended to have external patterns and were largely passive during the illness process [11]. Turkish studies on concepts of illness in Turkish cancer patients show similar results [4]. German patients often blame an unhealthy lifestyle for their illness [7], so it is helpful for them to actively seek rest and relaxation [11]. In a recent study on the causal attributions regarding cancer in German patients, environmental and psychosocial factors were the most frequently cited [12]. For Turkish patients, however, religion and faith were found to play important roles. These patients sometimes prefer supernatural causal attributions [13]. For example, some Turkish patients are of the opinion that the “evil eye” can cause accidents [13], or they may see the illness as a punishment from God and tend to behave passively [4,11,14], or they may actively try to deal with it, for example, by praying [15].

With regard to illness coping strategies, international studies demonstrate that Chinese cancer patients, for example, exclude their families from the illness process in order not to burden them [16]. Studies with Turkish patients show that religiousness is an important coping strategy [17]. On the one hand, religiousness can be seen as a protective factor in coping with illness, as the vast majority of studies show that religious people are less depressed [18,19] and anxious [19,20]. In addition, religiosity can also lead to the acceptance of disease-related changes [21]. On the other hand, a believer may also question his or her faith and feel abandoned by God [15]. Other studies on coping in Turkish patients show that they benefit from information searches [5,22], social support [23,24], and positive thinking [25] and, thus, these factors also influence disease prevention [26].

According to the Federal Statistical Office, migrants of Turkish origin make up the largest group of people with a migration background living in Germany, accounting for 2.8 million out of the total of 20.8 million [27]. Due to the Healthy Migrant Effect [28], it can be assumed that most migrants are still healthy at the time of immigration, because only healthy and resilient persons are able to resettle in Germany, a process that is associated with many challenges and difficulties. However, over the years, the social stress and risk factors they face in their new home country mean that they may also be affected by malignant illnesses, including some that are rare in their country of origin [28], and mortality rates rise accordingly [29]. Studies have shown that certain groups of migrants are at a higher risk of developing mental illnesses, such as depression, and also have a lower quality of life, which affects their ability to cope with the illness [30,31]. A German study also demonstrated that depressiveness is related to cultural adaptation; specifically, a high orientation towards the values of the host society and the culture of origin is associated with a low degree of depression [32]. It is therefore to be expected that migration status can have a variety of effects on the way a migrant copes with an illness such as cancer. However, despite the demographic situation in Germany, where 25.5% of the total population have a migration background [27], research on illness concepts and illness coping strategies is still scarce, particularly among oncology patients with a migration background. In particular, the above-mentioned studies have rarely drawn intercultural comparisons or established relationships between illness beliefs and coping strategies. The present study therefore aims to investigate the respective illness concepts and coping strategies as well as cultural differences regarding the two constructs between Turkish and German cancer patients by means of interviews.

## 2. Materials and Methods

The present study is a qualitative study. The data were obtained from guideline-based semi-structured interviews. The study was approved by the Ethics Committee of the Medical Faculty of Friedrich-Alexander University Erlangen-Nürnberg (file number: 232_14B). A written informed consent was obtained from each patient before the interview was conducted after a detailed explanation of the purpose and content of the study.

### 2.1. Sampling and Recruitment

Adult patients who were suffering from a malignant oncological illness at the time of the survey or who had suffered from this type of illness in the past (in adulthood) were interviewed. Half of the patients had a Turkish migration background; the other half were German citizens without a migration background. According to the Microcensus 2018, a migration background was defined as follows: “A person has a migration background if he or she or at least one parent does not possess German citizenship by birth.” [27]. The patients were recruited with the help of the psycho-oncological service of the Department of Psychosomatic Medicine and Psychotherapy at the University Hospital of Friedrich-Alexander University Erlangen-Nürnberg. Recruitment took place between July and December 2015.

### 2.2. Participants

A total of 22 persons were interviewed: 11 German and 11 Turkish patients. Of the German patients, 8 were male and 3 were female. They were aged between 24 and 57 years, with an average age of 46 years. Among the Turkish subjects, 9 female and 2 male patients, aged between 24 and 77 years, participated. Their average age was 59 years. Subjects had gone through different educational paths and worked in different professions. The majority of the Turkish patients had only completed primary school. Among the German interviewees, attainment of the secondary school certificate was the most common. Overall, the German patients had a higher level of education. Further socio-demographic, migration-related, and clinical data are presented in Table 1.

### 2.3. Instruments and Procedure

Based on guidelines, socio-demographic, migration-related, and medical data were initially collected. Subsequently, questions were asked on several topics (subjective concepts of illness, coping with illness, illness-related control beliefs, unmet psychosocial patient needs, attitudes towards the health care system/relationship with the doctor/barriers). For the present article, the topics “illness concepts” and “coping with illness” were selected. The question about the concepts of illness asked about the presumed cause of the illness. Five questions were used to examine how the illness was overcome, in which, among other things, the areas of emotions, family, and faith were examined in detail.

In order to prevent the results from being biased by language barriers, the interviews with the patients of Turkish origin were conducted in Turkish (except for one person who had an excellent knowledge of German language). All interviews in the respective groups were conducted by the same person. Interviews with the German patients and the Turkish-born study participant with excellent language skills were conducted by the last author (E.M.), and interviews of the 10 Turkish respondents were conducted by a Turkish-born psychologist from the psycho-oncology team. The interview duration was between 25 and 140 min for the German study participants and between 15 and 90 min for the respondents of Turkish origin. The interviews were recorded on a tape recorder and transcribed. The transcribed Turkish interviews were translated into German by a professional translator or by a former Turkish-speaking staff member. All German transcripts were completely checked for correct transcription by the first author and corrected if necessary. Sequences of approximately 10 min from each Turkish interview were compared with the transcript by a Turkish-speaking staff member.

### 2.4. Analysis

The evaluation was carried out according to the qualitative content analysis scheme developed by Mayring [33]. For this purpose, the two main deductively specified topics “illness concepts” and “coping with illness” were clearly defined and represented by anchor examples, and inclusion and exclusion criteria were specified in the form of coding rules. The transcribed interviews were independently examined by two evaluators (K.T. and E.M.) for statements that corresponded to the coding rules, and these statements were marked and assigned to the corresponding main topic. Atlas.ti version 7.5.18 software (Atlas.ti Scientific Software Development GmbH, Berlin, Germany) was used for this purpose. Finally, codes made by the two evaluators were checked for consistency and discussed and revised accordingly until a common consensus was reached. In the next step, all statements assigned to one of the two loci of interest were divided into thematic subcategories, and finally, the subcategories found were compared among German and Turkish patients. For the main topics, “illness concepts” and “coping strategies”, absolute frequencies were given for the subcategories identified. Due to the large number of aspects mentioned, the names of the individual subcategories of the coping strategies were summarized again under the main categories. All main and sub-categories were determined through several discussion rounds in consensus with the third co-author (Y.E.). In order to avoid errors in the assignments, a reverse check was also carried out by another person from the working group. This person was able to assign all subcategories determined by the text material exactly to the defined main categories and no redundant or missing main categories were identified.

## 3. Results

### 3.1. Illness Concepts

We identified a total of eight topics for the illness concepts, whereby the category “psychological causes” included several aspects. Differences could be observed regarding the frequency of reported illness beliefs between German and Turkish patients. There were also causes that were mentioned by only one of the two groups (Table 2).

The most frequent illness representation mentioned by the German patients was persistent stress.

“Then someone once explained to me that this could perhaps also be time-related. I separated from my husband in 2011; he died in a bicycle accident on New Year’s Eve 2012, and then I got the illness in 2014.” (German Patient. 1).

Toxins are another important attribution of causality for German patients. “Yes, maybe because I had started studying chemistry, and I might have inhaled something. Something harmful.” (German Patient. 5).

Toxins played no role at all in the group of migrants. Medical factors were also mentioned by some German patients as illness concepts. These factors included previous infections, for example Ebstein Barr.

Fate was also declared as a cause. All other less frequently mentioned illness beliefs can be found in Table 2.

If we look at the group of migrants, stressful life events were the most commonly reported individual explanation for cancer. Burdensome living conditions and stress were included under life events.

“Yes, the family problems I have experienced were the trigger. I was badly treated by my family and through the stress my body developed this illness.” (Turkish Patient. 11).

Traumatic experiences, which are described according to the ICD-10 as “a stressful event or situation of shorter or longer duration, with extraordinary threat or catastrophic proportions that would cause deep despair in almost everyone” [34], were treated as a separate category and were also frequently stated by patients of Turkish origin. For example, experiences of violence by the husband or the loss of a child were reported.

Aspects important to the Germans, such as medical factors as well as fate, were barely described as causes by Turkish patients. In contrast to the German patients, however, there was a new category of causes in Turkish patients, namely, the will of God. It also represents a very important illness concept in the migrants.

### 3.2. Coping Strategies

We identified eleven main categories, each with 2 to 10 different subcategories, as shown in Table 3: social support, activity, patient competence, fighting spirit/positive thinking, use of health services and alternative healing methods, lifestyle, emotional coping, cognitive coping, religious coping, spiritual coping, and (only for patients of Turkish origin) culture-specific methods of coping.

#### 3.2.1. Social Support

Social support is defined as any form of help from the social environment of the patient. It can be differentiated between practical and emotional support.

In both groups of patients, social support was found to play an important role and was often mentioned as an essential factor:

“In coping, what helps me? Psychological support, conversations with friends, my family being there, so that helps me.” (German Patient. 7).

What is different, however, is that among the Turkish patients, the neighbourhood was also included in the social environment.

“Everyone supported me. We have nine families here—with me, ten families. They didn’t leave me alone in the evening, or at midday, or in the morning.” (Turkish Patient. 8).

In addition, the type of support was also rather different between the groups. The friends and family of the German patients tended to focus on activating strategies, e.g., going for a walk or doing sports.

Encouragement and praying were predominant among the Turkish patients:

“Whenever I went for radiotherapy, I called everyone and asked them to pray for me. Because the mask was the hardest thing for me. If someone prayed, it went well. So, the next time, I said, ‘Let everyone pray for me’.” (Turkish Patient. 5).

#### 3.2.2. Activity

This category included physical activities, activities that are deliberately used for distraction, but also relaxation techniques or general rest and recreation. In addition, mental activities such as searching for information in books, on the internet, or through advice centres were also included.

In both patient groups, although more frequently among German patients, it was emphasized that physical activities such as cycling, swimming, running, or Nordic walking played important roles in coping with the illness.

“Yes, I also did a lot of sport in the autumn, i.e., moderate exercise.” (German Patient. 1).

“Sport is good for me, I always do sports. After rehabilitation, I do sports. I play sports twice a week.” (Turkish Patient. 10).

The intensification of hobbies, such as art, motorcycling, or cooking, was also highly valued in both patient groups. However, the spectrum was somewhat broader overall among the German patients.

Relaxation methods such as yoga, mindfulness, or progressive muscle relaxation were also predominantly mentioned by German patients.

In terms of searching for information, both groups used books or information material from the hospital to read about the clinical picture as well as about treatment methods. However, the internet was only mentioned as a source in the German group.

“I was doing research on the internet at that time and there was a seminar [Qi Gong for cancer patients] directly in the rehabilitation centre.” (German Patient. 2).

The Turkish patients, on the other hand, also asked medically educated friends, which was not stated for the German patients.

“[...] i.e., some friends who also studied medicine or were active in this field, and they were very supportive.” (Turkish Patient. 1).

#### 3.2.3. Patient Competence

This section summarizes statements that involve the patient’s autonomy and assertiveness. Communication with medical staff was mentioned as a particularly important aspect, especially for German patients.

“Yes. Because I want to understand that. And erhm, because I’m not a medical doctor, and if that’s too fast for me, particularly with technical terms, then I’ll just say, what does that mean for me translated? So, I ask them and write that down for me again.” (German Patient. 6).

In contrast to the Turkish patients, the Germans stated, as a further strategy, that they also specifically told their acquaintances and colleagues about the illness, i.e., dealt with it openly.

In addition, more German than Turkish patients reported that they have dealt openly with the consequences and continue to present themselves in public despite restrictions.

Turkish patients were characterized by a high level of compliance and a great deal of trust in the attending physician.

“If it weren’t for the doctors, I would be dead now. I had six months to live. It spread there, the doctor explained to me. If it weren’t for the doctors, I’d be gone now.” (Turkish Patient. 7).

For German patients, autonomy and also the assertion of their own interests were important aspects in dealing with the illness.

#### 3.2.4. Fighting Spirit

This refers to the will to actively approach and fight the illness or to think positively, taking emotional and cognitive aspects into account. In this category, no significant differences were observed between the two groups. Some German as well as Turkish patients reported strategies in the interviews that corresponded to a sense of the fighting spirit:

“I think that really helped me enormously, the story that I didn’t give up.” (German Patient. 4).

“But I have such a combative nature. It’s not because of the illness, it’s just... this is how I function in life.” (Turkish Patient. 1).

#### 3.2.5. Use of Health Services and Alternative Healing Methods

This includes all health care services offered by the clinics or health insurance companies. On the one hand, this includes psychological support and self-help groups; on the other hand, it also includes medical services such as medications that make it easier to deal with the illness, (e.g., antidepressants or tranquillisers), as well as medical rehabilitation, spa treatments, follow-up treatments, or gymnastics. Overall, the psychological offers and also the medical offers were used by both groups.

“I have had complementary conversations with a psycho-oncologist in the psychotherapy unit in the cancer counselling center in Nuremberg.” (German Patient. 2).

“I have seen doctors, therapies, and curers.” (Turkish Patient. 2).

In terms of frequency, it is noticeable that German patients reported taking advantage of these services in greater numbers (except for psychological and medical support) and also used a broader range of health care services overall. For example, only German patients were found to have attended fatigue counselling sessions or anti-stress groups or used telephone counselling.

#### 3.2.6. Lifestyle

An important point that concerns lifestyle is the change of diet. While Turkish patients tend to take additional vitamins, German patients try to change their previous diet, e.g., to avoid fast food or chemicals that might be in bottles made of polyethylene terephthalate.

It was also pointed out several times in both groups that the perception of nature, be it during a walk or simply by looking at it, helps to distract oneself from the illness.

It was even mentioned that one develops a new body consciousness, i.e., one perceives oneself and one’s body more consciously and respects it more.

“... what my body is worth to me... so I had such respect for the body, how it regenerated itself.” (Turkish Patient. 1).

Another difference is that only the German patients stated that they were increasingly doing themselves some good or indulging themselves.

“Especially when I am sometimes not feeling well or I am not feeling so great, I take a shower and then turn on my music on my mobile phone. Then, my mood somehow lifts again. Or when I get dressed and put on my makeup.” (German Patient. 8).

Furthermore, when asked about possible coping strategies, for many German patients, it was important to keep to a daily structure, and for some Turkish patients, it was important to adapt it to the new circumstances, for example, by allowing more breaks.

“So, I get up and make myself a really nice breakfast and keep to a daily rhythm like that.” (German Patient. 6).

“Or I also allowed myself to only be so active, but also in these... partly in these moments I withdrew, simply did nothing or did as much as I could—watched a film or did nothing at all, just looked out.” (Turkish Patient. 1).

Finally, in particular, the German patients stated that they had gained an appreciation of life through the illness.

#### 3.2.7. Emotional Coping

This category includes both positive emotions, such as hopefulness, joy, and pride as well as negative emotions such as fear, depression, denial, and anger. Each patient was specifically asked about his/her emotional handling of the illness, and both positive and negative strategies were mentioned in both groups.

Over the course of time, there were also some changes, and initial fears were transformed into confidence.

“But then at some point, I discovered this rhythm for me, where the body... and then I also converted that into a positive thing, that I knew that the body would manage.” (Turkish Patient. 1).

Another emotional coping strategy was acceptance.

“There was nothing I could do about it anyway. It was there. … accepted. I said, ‘why waste energy’, because I didn’t have that much energy there. I was more focused on, ‘How do I get out of this thing? ’” (Turkish Patient. 1).

“I took this for me, the illness is there, I took it, and I’m trying to make the best of it.” (German Pat. 11).

Comparing the two groups of patients, it is striking that Turkish patients were more likely than German patients to experience negative emotional processes such as denial, repression, discomfort, anxiety, and depression.

#### 3.2.8. Cognitive Coping

By cognitive coping we mean all strategies that have to do with perceptions and attitudes towards the illness. This includes, in particular, strategies such as fighting rumination, having plans/goals, decatastrophizing, rationalization, and relativization. These strategies were mentioned by both groups.

“Because I say, I am still young, I want to live, I want to marry, I want children, I would like to go back to my beautiful work.” (German Patient. 7).

Not thinking too much was also one of the cognitive coping strategies.

However, in addition, the targeted use of specialist knowledge or the search for role models or examples was a cognitive coping strategy that was used by both patient groups.

“And even then, through my profession, the knowledge of how one feels about chemotherapy, what the whole thing does to one.” (German Pat. 1).

“I always wanted to know what are my chances, or I asked friends or ... girlfriends who had this illness, who had overcome breast cancer, what they did. So I picked out good examples for me.” (Turkish Pat. 1).

#### 3.2.9. Religious Coping

Religious coping includes prayers, reading the Bible, or visiting a place of worship. Among Turkish patients, this category was one of the main categories; religious coping strategies played an important role for almost all patients. “But God‘s will will be done. If that is my destiny, then it will be so. I can’t make that determination. It’s up to God.” (Turkish Pat. 12).

#### 3.2.10. Spiritual Coping

Only among the German patients were spiritual methods, such as mantras or singing bowl rituals, observed.

“And I practice the [mantras] myself, too, but not like the Buddhists, not so extreme, but this thing helped me, too. This non-woman [monk in Buddhism] said that Buddhism wants all people to be happy, and if one chants this mantra over and over again, it has different meanings. Then, everything that contributes to my happiness comes into my life, for everyone involved.” (German Patient. 2).

#### 3.2.11. Culture-Specific Methods

These include, for example, various Turkish customs or herbal remedies.

However, many patients also said that they found a trip to Turkey particularly helpful for coping with the situation.

“My husband said, go to Turkey for three months, switch off, it will do you good. You look at other people and say, I am clearly better off than them. It’s true. I was on the Black Sea coast.” (Turkish Patient. 7).

The third culture-specific aspect was the visiting of Turkish doctors, which made it easier for some Turkish patients to deal with their illness.

## 4. Discussion

The aim of the study was to analyse and compare the illness concepts and illness coping strategies of German and Turkish patients with cancer in order to draw conclusions for clinical care.

In summary, similarities (e.g., stressful life events as triggers or social support as a coping strategy) but also clear differences could be identified. While German patients are often actively involved in the illness process, for Turkish patients, religious causes and coping strategies as well as culture-specific means are the main focus.

The illness concept that was most frequently reported by both patient groups were stressful life events. Environmental influences such as toxins were also an important illness representation among the Germans, while many patients of Turkish origin attributed their illness to the will of God or to a trauma. These findings are partially consistent with results from other studies on illness beliefs of Turkish migrants in comparison with the autochthonous population. A possible explanation for these differences may be based on the concept of health locus of control that has been widely studied in recent decades. It reflects the individual beliefs in internal or external causes of health outcomes (Rotter, 1954) [35]. In a study that examined the associations between health locus of control and depressive symptoms in a representative sample of native and non-native Dutch respondents, the Turkish minority group showed the highest scores on external locus of control from all investigated migrant collectives and the native Dutch [36]. Individuals from more collectivistic societies, such as Turkish societies, tend to have a higher external locus of control than persons enculturated in more individualistic societies. That means that they refer to (powerful) others to a greater extent than the latter ones. The frequent attribution of the illness to the will of God among the Turkish patients and in none of the German patients in our sample may reflect this cultural difference concerning the illness beliefs resulting from different health locus of control and/or a more pronounced religiosity among the Turkish patients. However, concerning the external health locus of control among Turkish migrants, there are also studies demonstrating no differences between persons of Turkish origin and the majority population. Schepker [37] examined a representative sample of Turkish adolescents of the second migration generation compared to their German counterparts and found no significant differences with regard to the levels of external health locus of control between both groups. Thus, the migration generation must be taken into consideration in investigations on illness beliefs in migrant collectives. In our study, almost all Turkish cancer survivors belong to the first migration generation, so a high degree of external health locus of control may be postulated as a possible explanation for the above mentioned differences concerning the illness concepts compared to the German patients. Future research should examine further and multiaxial constructs as potential variables contributing to intercultural differences in the context of illness concepts and coping strategies.

Environmental influences or an unhealthy lifestyle, which, in international studies, were found to play major roles in the development of illness in Turkish patients [4,38], were unimportant for the migrant group in our study. This could be partially related to the socio-demographic characteristics of our sample. While previous studies have demonstrated that Turkish men believed that air pollution and toxins in food were responsible for the genesis of cancer (4) [4], our sample consisted only of two men. Thus, it was too small to detect gender-specific differences in illness representations. Besides, prior studies in Turkish cancer patients indicated an association between education and illness beliefs, in such a way that persons with high education levels have attributed their illness to an unhealthy lifestyle. As many Turkish respondents in our investigation had low education levels, it might explain why the lifestyle did not play an essential role as a subjective illness belief.

Burdensome life events, such as conflicts or stress, were of primary importance for both groups in our investigation. In some of the Turkish patients, these burdens even fulfilled the criteria of a trauma. The fact that no traumas were named among the Germans could be related to the fact that German patients are in principle more open to psychological interventions [39] or are generally more active in coping and are therefore perhaps better able to deal with such experiences or have already processed them in such a way that they no longer play a significant role. Another explanation may be provided by the finding that migrants experience more traumatic events than the native population [40] so that they may serve for them as an explanation for the genesis of cancer. Overall, it shows that cancer patients see a close connection between physical and mental well-being and assume that their body’s own defences are greatly weakened by psychological stress. While the German patients stated medical factors as another important cause, the Turkish patients emphasized the influence of God. The finding that religion and faith play important roles among Turkish migrants has also been observed in other studies [4,14,41]. The German patients may be regarded as less religious than patients of Turkish origin. They, therefore, more often regarded “fate” as the cause when they could not find any other rational explanation for the genesis of their illness. The fact that medical factors and toxins dominated as causal attributions among German patients indicates that there are cultural differences and differences caused by the education level and the knowledge available. Patients with a lower level of education or a lower income tended to emphasize spiritual causes [1,12]. In addition, it must be taken into account that Turkish patients are not exposed to the same external factors in their home country and are thus confronted with new cancers after migration, for which they have little medical knowledge from their home country [41,42].

Regarding coping strategies, it was generally observed that both groups mentioned numerous and very individual strategies, which we summarized by eleven categories. Social support played an important role for both groups. However, the extent was much greater among Turkish patients. The Turkish patients stated that their families took on important roles in everyday life, such as cooking, while the German patients tended to generally spend time together. It is due to the Turkish understanding of family that sick relatives should be completely cared for and possibly even nursed [13], whereas German families rather try to distract the patient from his illness. These cultural differences may result from the fact that the more collectivistically oriented Turkish patients live in cohesive family structures that provide social support but also dependence from family members, while autonomy and responsibility are central values for the more individualistically oriented German patients [43]. The Turkish culture can be regarded as a socio-centric one in which the whole family or even community is engaged in the healing process [44].

Franz et al. [11] also showed that Turkish patients more often play a passive role in coping with their illness. They benefit from the secondary gain from the illness, i.e., the support of their family. A social network offers emotional, informal, and active support [24]. Other studies have also emphasized the essential role of social support in coping with illness [5]. This study did not show that women, in particular, tend to distance themselves from their families because they can no longer play their role as caregiver. In addition, Turks attribute their illness largely as being God-given and, therefore, they regard themselves as having little influence on the illness which also explains why they use less active coping strategies [4]. Although they reported fewer activities in the distraction category than the German patients, it was still important for them to be informed about their illness. Thus, although Turkish patients assume that they cannot actively influence a God-given illness, the search for knowledge about the illness reflects a need for control. In line with results from previous studies, Turkish patients showed great confidence in medicine and their doctors in this regard [5,28], while German patients were also more likely to be informed by books or acquaintances. On the one hand, this may be related to a language barrier. On the other hand, education and the individual character of a person, which is also culturally determined, also play important roles, as demonstrated by Kav et al. [5].

Patients with a higher socioeconomic status tended to avoid ineffective coping strategies [24,45] and usually had more prior medical knowledge [17]. The trust of Turkish patients in classical medicine is also reflected in the current study by the finding that the Turkish patients asked more about pharmacological therapies. However, in contrast, they used alternative services of the health system, such as sports groups, less. This may also have been caused by difficulties in understanding, as well as by a different way of dealing with the body or cultural barriers. This should, however, be taken into account to ensure adequate patient care. The importance of culture-specific coping strategies is reflected in the fact that many Turkish patients reported such strategies. Franz et al. [11] also demonstrated that Turkish patients are more likely to believe in a chronic course, at least in the case of mental illness. This can serve as an explanation as to why negative feelings such as depression and sadness are particularly important in the emotional coping of Turkish patients. This was also shown by Özkan et al. [10]. Furthermore, the negative emotional coping of the patients of Turkish origin in our study may be influenced by the external locus of control. Van Dijk et al. [36] have demonstrated an association between high scores in external locus of control and high levels of depressive symptoms in the multi-ethnic Dutch population including Turkish migrants. In addition, the negative emotions are intensified by the fact that the patients may doubt their faith or ask themselves why God has imposed this illness on them and feel helpless [13]. Overall, Turkish patients found it difficult to deal with the illness openly, which can be explained, for example, by the cultural obligation to remain healthy [13]. In addition, cancer is a taboo subject, especially for traditionally minded Turks, and cancer is a word that should be avoided as much as possible [17]. As was also demonstrated by Bermejo et al. [7], German patients had tried to actively influence their illness. For them, this also included doing something good for themselves. Since some of the Turkish patients saw illness as a punishment, it is understandable that they did not mentioned this as a strategy, although positive thinking or distraction through positive activities is crucial for quality of life [25].

In contrast to Turkish patients, Germans were found to be more interested in alternative healing methods regarding the services of the health system. This could certainly also be related to their level of education or that the German patients named medical factors such as toxins or infections as the cause of their illness and therefore wanted less chemotherapy, for example. They are also less religiously influenced than the Turkish patients and therefore more open to different spiritual practices. An acceptance of the illness was found in both groups. For most Turkish patients in our sample, however, this seemed to be due to thinking it was God-given and unstoppable, whereas German patients saw it as a motivation to change their lives or to be more attentive to their own needs and to appreciate the life and enjoy the moment. Other studies have shown that Turkish patients also use other coping strategies, such as changing priorities in life, but are still guided by basic religious ideas such as “Shukran” (gratitude) [15]. Psycho-oncological counselling was found to be an important support mechanism for many German patients. Psychological support in the mother tongue also played an important role for Turkish patients, as reflected in other studies [28]. In addition to coping with the illness, the use of psycho-oncological care is certainly also helpful in dealing with the stressful life events mentioned as causes. However, religious coping was found to be one of the most important strategies in dealing with cancer for Turkish patients [4]. For them, God’s will is not only the cause of the illness, but they also see God as a source of support in coping with it, and through prayer, they receive the necessary strength [17].

### Strengths and Limitations

The most important strength is that the present study is the first to compare illness concepts and illness coping strategies between German and Turkish cancer patients. Another strength is the fact that, in order to avoid a linguistic bias, the interviews with the Turkish patients were conducted in Turkish. However, the interviews were conducted by two different people, so despite a detailed discussion of the procedure, it cannot be excluded that the subjective interview style could have impaired the objectivity of the performance. For example, the Turkish interviews were shorter. Since there was only a small number of Turkish patients in the clinic, the samples of only 11 persons each are relatively small. Furthermore, the patients were not matched in terms of socio-demographic (sex, age and education), medical, or life history (e.g., traumatisation) data prior to the study. Finally, as is the case with all retrospective studies, a memory bias cannot be ruled out. Future studies in larger, representative, and matched (regarding sex, age and education) patient samples are therefore necessary.

## 5. Conclusions

The abovementioned results have clear clinical implications, especially for the treatment of Turkish patients. In addition to considering the language barrier, culture-specific aspects such as the handling of the body and the importance of religion and family should be taken into account. Migration does not mean that concepts and patterns of behaviour change fundamentally. Information and educational material should therefore always be available in Turkish for cancer survivors of Turkish origin, in particular, for first-generation migrants. In addition, the level of education should be taken into account and information should also be presented, e.g., through pictures. Health system services such as sports groups should specifically include groups for Turkish patients, and families should be involved in treatment at an early stage or should be considered as an important anchor when it comes to delivering difficult news. Although Turkish patients are very compliant, they should also be supported with their patient competence, as they do not always demand it, as German patients often do. Finally, individual concepts should also be asked about in all patient groups and integrated into the treatment plan, since, for example, psychological support for a traumatized patient contributes significantly to improving his/her ability to cope.

In addition to the specific offers for patients, it is also important to sensitize the health professionals sufficiently for the cultural characteristics. Essential components of this cultural training are, on the one hand, advanced training courses on the topics of illness concepts and strategies, which in the best case are held by employees who belong to the respective culture. On the other hand, regular supervision and discussions in a multidisciplinary team can help to ensure that the patient and his/her cultural needs are considered in all areas of treatment.

## Figures and Tables

**Table 1:** Socio-demographic and clinical characteristics of German and Turkish cancer patients.

ID	Gender	Age	Highest School Degree	Marital Status	Type of Cancer	Year of Initial Diagnosis
1-GE	female	48	Secondary school	widowed	Breast cancer	2014
2-GE	female	57	Secondary school	divorced	Jaw, interior, oral cavity carcinoma	2011
3-GE	female	39	University/Promotion	unmarried	Breast cancer	2013
4-GE	male	55	Secondary school	married	Skin cancer	2009
5-GE	female	45	University/Promotion	married	Brain tumour	2006
6-GE	female	49	High school	unmarried	Breast cancer	2015
7-GE	female	24	Secondary school	unmarried	Stomach cancer	2015
8-GE	female	54	Secondary school	married	Colon cancer	2015
9-GE	male	52	Secondary school	married	Leukaemia	2015
10-GE	female	48	Qualified secondary modern school certificate	married	Lymphoma	2015
11-GE	male	35	High school	married	Lymphoma/Leukaemia	2015
1-TR	female	53	University	unmarried	Breast cancer	2009
2-TR	female	59	Primary school	married	Sarcoma	2012
3-TR	female	69	No graduation	married	Pharyngeal carcinoma	n/a
4-TR	female	64	Primary school	widowed	Uterus cancer	2011
5-TR	female	63	Primary school	married	Spinal tumour	n/a
6-TR	male	77	No graduation	married	Prostate cancer	2018
7-TR	female	61	Primary school	married	Colon cancer	2001
8-TR	female	75	n/a	widowed	Colon cancer	2018
9-TR	male	24	Vocational school	unmarried	Lymphoma	2013
10-TR	female	56	Primary school	married	Thyroid cancer	2014
11-TR	female	49	Secondary school	divorced	Breast cancer	2010

GE = German; TR = Turkish; n/a = not available

**Table 2:** Illness concepts of German and Turkish cancer patients.

Illness Concepts	German Patients (*n* = 11)	Turkish Patients (*n* = 11)
	N *	N *
Stressful live events	6	5
Environmental influences	5	0
The will of God	0	4
Trauma	0	4
Medical factors	3	1
Fate/bad luck/normality	3	1
Health behaviour	1	1
Psychological causes	2	3
-Not processing important life events	0	2
-Guilt/punishment	1	0
-Lack of self-care	1	0
-Family stress caused by misconduct by the husband	0	1

* Multiple answers possible.

**Table 3:** Illness coping strategies of German and Turkish cancer patients.

Categories	Subcategories	Ger. Pat. N *	Tur. Pat. N *
**1. Social support**	-Emotional support (e.g., phone calls, visits, good coaxing, conversations)	11	10
	-Practical support (e.g., help in the household, accompaniment to examinations)	8	6
**2. Activity**	-Activity (e.g., (moderate) sport, walking, painting, working)	11	5
	-Distraction (e.g., watching movies, listening to music, reading, spending time with family)	9	3
	-Searching for information (e.g., internet, books, health insurance, counselling centre, doctors)	7	4
	-Relaxation/imagination techniques/mindfulness	6	2
	-Lying down/recreation/rest	4	2
	-Travel	3	1
	-Other (e.g., leave the clinic for an hour, keep tally sheets for chemotherapy)	4	3
**3. Patient competence**	-Openly dealing with the illness and its consequences	9	2
	-Demarcation (towards medical staff/burdensome discussions/calls/visits/persons)	7	2
	-Making good contact with doctors/having confidence in doctors	6	3
	-Compliance (e.g., going to check-ups)	1	6
	-Assertiveness/pursuit of own interests/self-confident communication with doctors (e.g., asking questions)	5	3
	-Preparing for doctor’s appointments/writing down the doctor’s answers/wanting to be kept up to date with the illness	1	2
	-Autonomy (e.g., changing ostomy bags independently)	0	2
	-Other (e.g., getting a second opinion, exchanges with other patients)	3	2
**4. Fighting spirit/positive thinking**	-Fighting attitude towards cancer/positive thinking	8	6
**5. Use of health services and alternative healing methods**	-Psychological support (e.g., psycho-oncological consultations, outpatient psychotherapy)	6	7
	-Medical support	4	6
	-Medication (e.g., for sedation, painkillers, antidepressants)	2	6
	-Gymnastics/physiotherapy/rehabilitation sports	5	2
	-Herbs/homeopathy/naturopathy	4	1
	-Rehab/cure/follow-up treatment	3	3
	-Social services/specialist medical services/telephone counselling	3	0
	-Self-help groups/cancer counselling centres/fatigue consultation	2	0
	-Vitamin tablets	0	2
	-Other (e.g., speech therapy, massages, human genetic testing, kinesiology)	2	1
**6. Lifestyle**	-Openness/trying out new things/flexibility	7	1
	-Self-care/being more attentive to one’s own needs/indulging oneself /buying something (e.g., electric bike, ergometer)	6	3
	-Appreciation of life/enjoying beauty/nature/the moment	6	1
	-Maintaining a daily structure/leading a normal life	6	1
	-Nutritional change/healthy nutrition/giving up harmful/unhealthy things (e.g., plastic, fast food)	4	3
	-Adaptation to the daily form/physical fitness	4	2
	-Altruism	3	1
	-Other (e.g., naturism, open contact with the body, accepting help, new body awareness)	4	0
**7. Emotional coping**	-Hopefulness/Confidence	7	6
	-Denial/repression/not telling anyone about cancer/pretending false feelings	2	5
	-Struggling/grief/crying	3	4
	-Joy/happiness/feeling proud/smiling	3	2
	-Acceptance of the illness/its consequences	3	1
	-Other (e.g., anxious/depressive coping, anger at cancer, naming emotions, comforting oneself, not wanting to be pitied)	3	3
**8. Cognitive coping**	-Rumination	4	6
	-Fighting the rumination/not worrying too much/not thinking too much	4	5
	-Plans/goals/thinking of important people	5	2
	-Rationalization/relativization by comparison/decatastrophication	1	3
	-Humour	3	0
	-Use of specialist knowledge/bicultural knowledge	2	1
	-Waiting for aftercare/”one after the other”	1	2
	-Other (e.g., focusing on recovery, saying tumour instead of cancer, looking for good role models/examples)	2	3
**9. Religious coping**	-Prayers (alone/with others/prayers requested from others)	3	6
	-Devotion to the will of God/acceptance of the illness as the will of God	0	5
	-“Pure heart”/have done no evil/have forgiven	0	4
	-Other (e.g., consolation in faith, conviction that God has healed/sent help, reading the Bible, going to the mosque)	2	7
**10. Spiritual Coping**	-E.g., as meditation, mantras, shamanism, singing bowl rituals	3	0
**11. Culture-specific means of coping**	-Turkish customs/remedies/products	-	3
	-Travel to Turkey	-	3
	-Consulting Turkish doctors	-	2
	-Turkish friends	-	1

Ger. Pat. = German patients; Tur. Pat. = Turkish patients; * Multiple answers possible
